# The Development and Validation of a Novel Smartphone Application to Detect Postural Instability

**DOI:** 10.3390/s25051505

**Published:** 2025-02-28

**Authors:** Shirin R. Hussain, W. Geoffrey Wright

**Affiliations:** Department of Health & Rehabilitation Sciences, Temple University, Philadelphia, PA 19122, USA; tul13885@temple.edu

**Keywords:** smartphone technology, validation, inertial measurement units, postural stability

## Abstract

Traditional assessments of balance and postural control often face challenges related to accessibility, cost, subjectivity, and inter-rater reliability. With advancements in technology, smartphones equipped with inertial measurement units (IMUs) are emerging as a promising tool for assessing postural control, measuring both static and dynamic motion. This study aimed to develop and validate a novel smartphone application by comparing it with research-grade posturography instruments, including motion capture and force plate systems to establish construct- and criterion-related validity. Twenty-two participants completed the quiet stance under varying visual (eyes open—EO; eyes closed—EC) and surface (Firm vs. Foam) conditions, with data collected from the smartphone, force plate, and motion capture systems. Intraclass correlation coefficients (ICCs) and Pearson correlation coefficients assessed the reliability and validity for all outcome measures (sway area and sway velocity). The results demonstrated reliability, with strong validity between the devices. A repeated-measures ANOVA found no significant differences between the devices. Postural outcomes revealed the significant main effects of both the visual (EO vs. EC) and surface (Firm vs. Foam) conditions. In conclusion, the study demonstrated the validity, sensitivity, and accuracy of the custom-designed smartphone app, offering the potential for bridging the gap between at-home and clinical balance assessments.

## 1. Introduction

Human balance involves maintaining and restoring balance to a state of equilibrium and upright orientation [1]. The spinal and supraspinal neural circuitry involved in this process [2] receive constant sensory feedback from the vestibular, visual, and somatosensory systems, which help to guide the motor responses for postural stabilization. Even during the unperturbed quiet stance, small postural fluctuations reflect the motor activity that is needed to maintain the body’s center of mass safely within its base of support [3]. There are many reasons for postural instability, including age and metabolic and neuromuscular dysfunction. Changes in postural stability may be subtle and are often compensated in the routine activities of daily living (ADL), as our bodies are constantly adapting and reweighting sensory inputs to maintain balance. However, as fall risk factors increase, the likelihood of postural instability also increases, so it is often only after a fall has occurred that postural deficits are noticeable. This reactive approach to falls leads to considerable medical costs. In the US alone, the costs related to falls are expected to exceed over $100 billion USD annually by 2030 [4].

Traditionally, balance and postural control have been assessed through clinical tests, such as the Romberg test (subjective) and Timed-Up and Go (TUG), or instrumented or computerized tests, such as the Sensory Organizational Test [5,6,7], which may include kinetic (e.g., center of pressure: COP) and kinematic (center of mass: COM) metrics to derive variables such as postural sway area and sway velocity [3,8]. In research settings, criterion measures for the COM and COP include motion capture systems and force plates; however, each has its own limitations. Most notably, the qualitative tests can be subjective and/or have low inter/intra-rater reliability, while the quantitative methods require expensive equipment, a trained clinician, and dedicated space. Together, these factors limit the accessibility of objective fall risk detection and, as a result, may not be recognized until it is too late [9].

In the last decade, advances in smartphone technology have created the potential for balance screening tools because of built-in inertial measurement units (IMUs) with the capacity to measure six-degrees-of-freedom movements with great accuracy. This has led to novel approaches to objectively measure balance with minimal expertise [10,11,12,13,14,15,16,17]. As of 2023, it is estimated that 90% of the US population owns a smartphone device, making them a convenient and accessible tool [18]. However, to make smartphone IMUs effective for clinical applications, it is crucial to evaluate their validity, reliability, and sensitivity in comparison to gold-standard laboratory and clinical assessments.

To date, few studies have validated smartphone IMUs for posturography against gold-standard instrumentation (e.g., force plates, motion capture systems, and accelerometers) [12,15,19,20,21,22,23], and those that have vary considerably in approach (e.g., postural tasks, device placement, and orientation), the equipment used, and the populations tested [19,20,21,22,23,24].

Given the increasing consensus that smartphone-based inertial measurement units (IMUs) can serve as effective tools for collecting human movement, a considerable body of research has emerged over the past decade to assess their functionality. Ozinga et al. used an iPad application and found it was significantly correlated (r = 0.89–0.99) with their 3D motion capture measurements during the quiet stance [25]. Kosse et al. evaluated both gait and standing posture with an iPod Touch against standalone accelerometers, and found strong correlations (r = 0.85–0.99) and a good-to-high degree of reliability (ICC = 0.78–0.99) [26]. Cerrito et al. evaluated their Android application against force plate measurements on a sit-to-stand test, which also showed strong correlations (r = 0.86–0.93) and reliability (ICC = 0.42–0.96) [27].

More recently, Grouios et al. assessed the validity and reliability of acceleration data across three smartphones of different makes and models against a motion capture system during various gait trials. The study demonstrated no statistically significant differences in mean acceleration values between each device. Additionally, for the smartphones evaluated, the study demonstrated that the devices were both valid and reliable for estimating acceleration when compared to an established gold standard [28]. These findings align with other studies that have compared the performance of IMU sensors in smartphones to gold-standard instrumentation. Frechette et al. compared their Android application with research-grade accelerometers and found strong correlations between the outputs from both devices across various balance tasks (ρ = −0.75 to 1.00; *p* ≤ 0.01) [19]. Hsieh et al. reported moderate-to-high correlations (ρ = 0.42–0.81; *p* < 0.01–0.05) when comparing a force plate system to an Android application during quiet stance [21]. These results are promising, as the outcome of a smartphone application that accurately measures balance could meet an essential need for high fall-risk populations who have limited access to clinical resources.

Using an affordable, portable, easy-to-use device, such as a smartphone, opens the potential for at-home or clinical assessments without the initial need for a clinician. The purpose of this study was to develop a novel smartphone application and validate it relative to research-grade posturography instrumentation (i.e., motion capture and force plate systems). Demonstrating the construct- and criterion-related validity of a smartphone application relative to gold-standard posturography devices will help substantiate its potential for clinical applications.

## 2. Materials and Methods

### 2.1. Participants

A total of 22 participants (11 males and 11 females; age range 20–60 years.; mean age = 43.0 ± 13.1 yrs.), free from any pre-existing condition that could affect their ability to balance normally, were tested in a single-session repeated-measures design study. Ethical approval was granted by Temple University’s Institutional Review Board and all participants provided written informed consent before engaging in the research.

### 2.2. Instrumentation and Data Processing

Quiet stance was measured using (1) a smartphone device (Apple iPhone 14, Apple Computer Inc., Cupertino, CA, USA), (2) a 7-camera motion capture system (Motion Analysis System, Inc., Santa Rosa, CA, USA), and (3) a force plate (Bertec corporation, Columbus, OH, USA). In each test trial, postural data were collected simultaneously from these three electronically synchronized devices. To synchronize each of the three devices during data collection, the smartphones were linked to a local desktop over WIFI (IP address) using a TCP/IP protocol and a custom Python script (Version 3.12). A Macro Recording application (Macro Recorder, Bartels Media Inc., Version 2.0.79) was used to send a signal to the motion capture system, force plate system, and smartphone device’s user interface to begin each data collection trial. To the authors’ knowledge, this is unique to the present study, as no other smartphone validation studies have employed this synchronization technique. Previous research has typically achieved synchronization through a post-processing methodology.

#### 2.2.1. Smartphone Application

**Figure 1:**
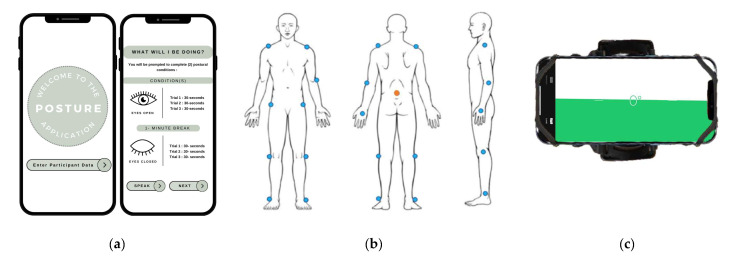
(**a**) The smartphone application user interface was controlled remotely by the experimenter. (**b**) Motion capture markers were placed on the participant’s body in the anterior and posterior positions. The orange dot illustrates the marker located on top of the smartphone device, which was attached at the L5 region. (**c**) For the greatest sensitivity, the smartphone was oriented horizontally and was secured to the participant using a belted phone holder. Orientation was confirmed using Apple’s preloaded Measure application to confirm the spirit level prior to data collection.

A smartphone application was developed for an iPhone 14 using XCODE (Apple Computer Inc., Cupertino, CA, USA), Apple’s integrated development environment for Mac iOS. A single iPhone 14 was used to collect kinematic postural sway metrics [29]. The built-in IMUs in the smartphone are capable of detecting movement in 6-degrees-of-freedom, with the primary axes of measure for this study being linear displacement along the intrinsic axes of the smartphone, defined as *x* (width), *y* (length), and *z* (thickness) [30]. Pilot testing revealed that the iPhone’s *z*-axis maintained the same accuracy regardless of the orientation, and thus was used to collect anterior–posterior (AP) displacements. However, there were significant differences in the IMU sensitivity for the other 2 axes. When measuring medial–lateral (ML) postural motion, the smartphone’s correlation with the research-grade motion capture measurements was significantly lower for *x* (r = 0.12–0.71; *p* < 0.05) than *y* (r = 0.64–0.89; *p* < 0.05). Therefore, the smartphone was oriented horizontally (Figure 1c), such that the *z*- and *y*-axes were the primary axes used to estimate the COM movement.

Prior studies using IMUs have attached the sensor to the lower back to estimate the motions of the center of gravity [31,32,33,34]. The smartphone was positioned at the participant’s L5 to approximate the COM position [24,35], and securely fastened to the waist via a modified running belt (VUP Phone Holder) (Figure 1c). The phone belt served the following two purposes: (1) standardizing the location of the smartphone and (2) mitigating the horizontal component of the gravity vector due to any tilt of the device that would affect the accelerometers in the horizontal measurement axis. The smartphone level was determined using the Measure application (a preloaded application in iOS) to ensure the absence of tilt prior to data collection (Figure 1c). In addition, any constant bias in the signal due to the gravity component was subtracted out during post-processing by normalizing the data set (i.e., subtracting the average of the samples in a trial from each individual sample in the trial). Ghislieri et al. [36] point out that very little to no information is provided in the literature about how the misalignment of sensor axes might affect the measurements, particularly for the influence of gravity on the ML and AP axes. However, because even small misalignments could lead to measurement errors, we followed their guidance of using a more rigorous approach to orientating the sensor axes in relation to the global reference frame.

The accelerometers from the smartphone sampled at an average of 100 Hz. The raw acceleration data from the device were collected using the Application Programming Interface [29] provided by Apple. During post-processing, high-frequency noise was filtered from the data with a 4th-order low-pass Butterworth filter using a custom MATLAB R2022a script.

#### 2.2.2. Motion Capture (MC)

A seven-camera motion capture system (Motion Analysis System, Inc., Santa Rosa, CA, USA) was used to collect body kinematics. Eleven reflective passive markers were placed on each participant in the following locations: the left and right acromion processes, the left elbow, the left hand, the left and right anterior superior iliac spine (ASIS), the left and right lateral patella, the left and right lateral malleoli, and the L5 (placed on top of the smartphone; see Figure 1b). This model assumes the body is a single-link inverted pendulum [37], and the lumbar marker was used to approximate the COM position [24,31,32,33,34] to derive the postural metrics for the analysis. The position from the motion capture system was sampled at an average of 100 Hz and processed with a 4th-order low-pass Butterworth filter using a custom MATLAB script.

#### 2.2.3. Force Plate (FP)

A force plate with Digital Acquire 4.1.20 software (Bertec corporation, Columbus, OH, USA) was used to collect the kinetic center of pressure (COP) data in the ML and AP directions. The COP time-series data from the force plate were sampled at an average of 1000 Hz. They were then exported and processed with a 4th-order low-pass Butterworth filter at a cutoff frequency of 10 Hz using a custom MATLAB script [3]. The COP was transformed into a COM estimate using an established single-link inverted pendulum model in which a zero-phase low-pass filter was applied to the postural data [3,38].

#### 2.2.4. Postural Formulae

Using the normalized and filtered data, the sway area was derived using a Principal Component Analysis (PCA), where a = the maximum and minimum of the major axes and b = the maximum and minimum of the minor axes [39,40]. The major and minor axes were determined based on the AP and ML displacements of the L5.

Sway velocity was derived using the following equation:

(1) $$Sway\;Velocity=\frac{\sum_{i=1}^{n}\sqrt{\frac{{\left(z_{\left(i+1\right)}\;-\;z_{i}\right)}^{2}+{\left(y_{\left(i+1\right)}\;-\;y_{i}\right)}^{2}}{t_{\left(i+1\right)}\;-\;t_{i}}}}{n}$$

where *z_i_* and *z*_(*i*+1)_ are the AP coordinates of two consecutive samples, and *y_i_* and *y*_(*i*+1)_ are the ML coordinates of the two consecutive samples. The Pythagorean distance between two consecutive samples was calculated and then divided by the time (*t*) between the samples (*t*_(*i*+1)_–*t_i_*) to obtain the instantaneous velocity between the consecutive samples. The sway velocity was then calculated from an average of these instantaneous velocities. The force plate data were downsampled from 1000 Hz to 100 Hz after filtering to synchronize them with the smartphone and motion capture systems. This ensured the consistent timing across all data sources, thus avoiding any discrepancies in the sampling rates.

The somatosensory ratio was derived using the following equation based on the postural outcomes (sway area and sway velocity) visual conditions:

(2) $$Somatosensory\;ratio=\frac{\left(eyes\;closed\right)}{\left(eyes\;open\right)}$$

#### 2.2.5. Postural Task and Protocol

Participants were instructed to stand still and upright with their feet hip-width apart and their hands crossed over their chest, wearing a modified running belt around the waist to securely hold the iPhone in place (Figure 1c). The postural task focused on the instrumented version of the Romberg test, measuring each participant’s COP using the force plate system and the COM using the motion capture system. These instruments served as our criterion measure against which the custom smartphone application was compared.

**Figure 2:**
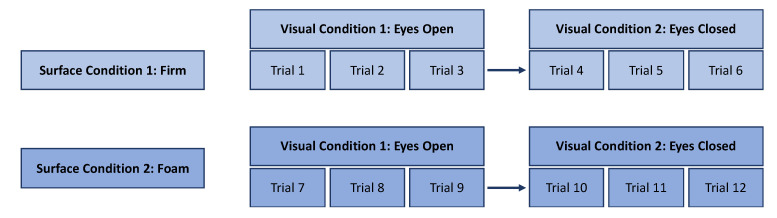
The order of testing for each participant. Firm surface and eyes open visual conditions were performed first during each data collection session. A total of twelve 30-s trials were administered to each participant.

**Figure 3:**
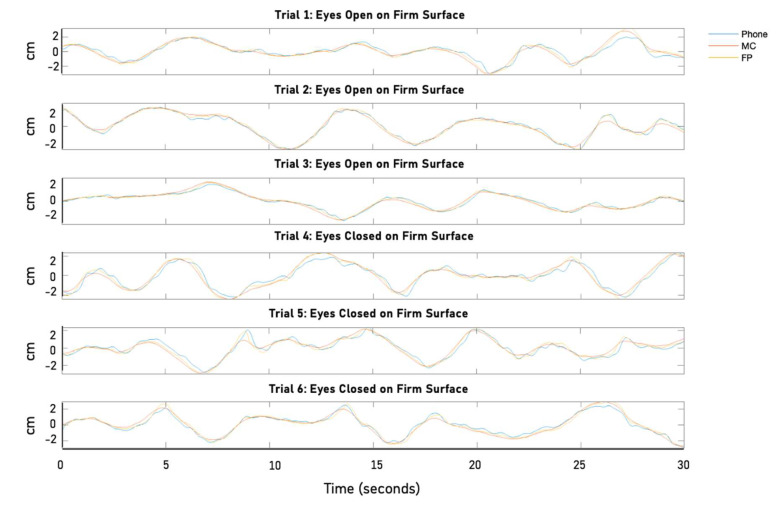
Time-series plots illustrating the postural movement data (AP sway) collected using three synchronized instruments from one representative participant tested in trials 1−6.

Postural data were collected during a single 60-min session, with each participant performing all 12 trials in the same prescribed order (Figure 2). During the session, the participants were barefoot, with their feet positioned a hip-width distance apart, while standing on a firm and then a foam surface. All of the trials were collected while the participants were standing on a force plate (or with foam placed on top of the force plate), while collecting the COP, 3D translation of the L5 marker, and 3D translation of the smartphone attached to the waist. Three 30-s trials for each visual condition were collected, with a 1-min break between eyes open (EO) and eyes closed (EC), for a total of 6 trials per surface condition (Figure 3). Testing both visual (EO and EC) and surface (Firm and Foam) conditions in the research design provided a gauge of the sensitivity and accuracy of the smartphone sensors during highly stable conditions (e.g., EO–Firm) and potentially unstable conditions (e.g., EO–Foam) for validation purposes.

#### 2.2.6. Statistical Analysis

To address the criterion validity, Pearson correlation coefficients were calculated by comparing the postural variables (sway area or sway velocity) between the gold-standard instruments and the novel smartphone application. Additionally, time-series correlations were calculated to assess the moment-to-moment variations in the positional data of the smartphone and motion capture system across visual and surface conditions for both the AP and ML axes. Correlation coefficients of 0.1 were considered weak, 0.3 were considered moderate, and 0.5 to 1.0 were considered strong [41]. To gain further insight into the individual spread of the measurement error between the smartphone application and each of the gold-standard instruments, the postural outcome measurements of the sway area and velocity were analyzed using Bland–Altman plots. In each plot, the average value for each pair of measurements was plotted against the mean difference between the two values of the two measurement devices for the individual data. In addition, the upper and lower limits of agreement, as 1.96 x standard deviation, were calculated for the two devices [42].

A repeated-measures ANOVA was conducted to examine the effects of the surface (Firm vs. Foam), device (MC, Phone, and FP), and visual conditions (EO vs. EC) on the sway area and sway velocity dependent variables. Mauchly’s test was used to evaluate the sphericity. If the sphericity was violated, then the degrees of freedom were adjusted using a Greenhouse–Geisser correction. A *p*-value of less than 0.05 denoted the presence of a statistically significant difference.

A series of intraclass correlation coefficients (ICCs) were calculated to examine the validity and reliability of the smartphone device. To assess the test–retest reliability, ICC (3,1) estimates and their 95% confidence intervals were calculated. This was based on a mean-rating (*k* = 3), absolute-agreement, and 2-way mixed-effects model that was applied to the measurement outcomes (sway area or sway velocity) for each of the 3 trials collected per device (MC, Phone, and FP). ICC (3,*k*) estimates and their 95% confidence intervals were calculated based on a mean-rating (*k* = 3), absolute-agreement, and 2-way mixed-effects model for the somatosensory ratio of the measurement outcomes (sway area or sway velocity) of the novel device when compared to the motion capture or force plate system. The ICC (3,*k*) was calculated based on a mean-rating (*k* = 3), absolute-agreement, and 2-way mixed-effects model to examine the averages of measurement outcomes (sway area or velocity) across the three devices. ICC values below 0.5 were interpreted as demonstrating poor reliability, values ranging from 0.5 to 0.75 reflected moderate reliability, values between 0.75 and 0.9 indicated good reliability, and values above 0.90 signified excellent or a high degree of reliability [41].

Data post-processing and statistical analysis was performed using MATLAB R2022a (Mathworks Inc., Natick, MA, USA) and SPSS version 29 (IBM Corp, Armonk, NY, USA).

## 3. Results

The demographic information of all participants is presented in Table 1.

**Figure 4:**
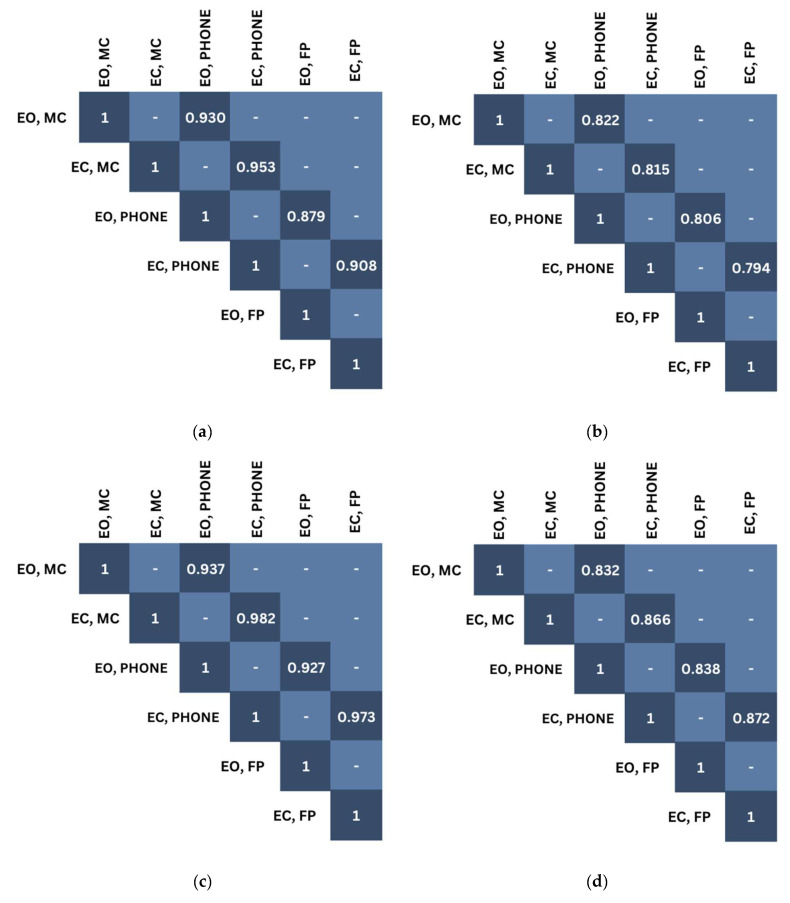
Pearson correlation applied across the group mean postural variables: (**a**) Firm sway area; (**b**) Firm sway velocity; (**c**) Foam sway area; (**d**) Foam sway velocity.

The novel device demonstrated strong correlations with the motion capture and force plate systems in all of the visual and surface conditions for both the sway area and sway velocity postural outcome measures, as shown in Figure 4. Under foam surface conditions, the r-values appear to increase with both the sway area (r = 0.93–0.98; *p* < 0.05) (see Figure 4c) and sway velocity (r = 0.83–0.87; *p* < 0.05) (see Figure 4d) when compared to firm surface conditions (r = 0.88–0.995, *p* < 0.05; r = 0.79–0.82, *p* < 0.05) (see Figure 4a,b). The results demonstrate that, as the conditions increase in difficulty (i.e., EO–Foam and EC–Foam), the correlations strengthen.

The AP linear displacements of both systems demonstrated strong correlations across both the visual and surface conditions (r = 0.857–0.913; *p* < 0.001). The ML linear displacements of both systems showed moderate-to-high correlations across the visual and surface conditions (r = 0.397–0.707; *p* < 0.05) (Table 2). While the ML positional correlations were statistically significant, they were comparatively weaker than the AP positional correlations, suggesting a more variable relationship between the motion capture system and smartphone’s ML positional time-series data across the visual and surface conditions. As the conditions increased in postural difficulty (i.e., EO–Foam and EC–Foam) and the sway increased, the ML time-series positional correlations increased in strength.

**Figure 5:**
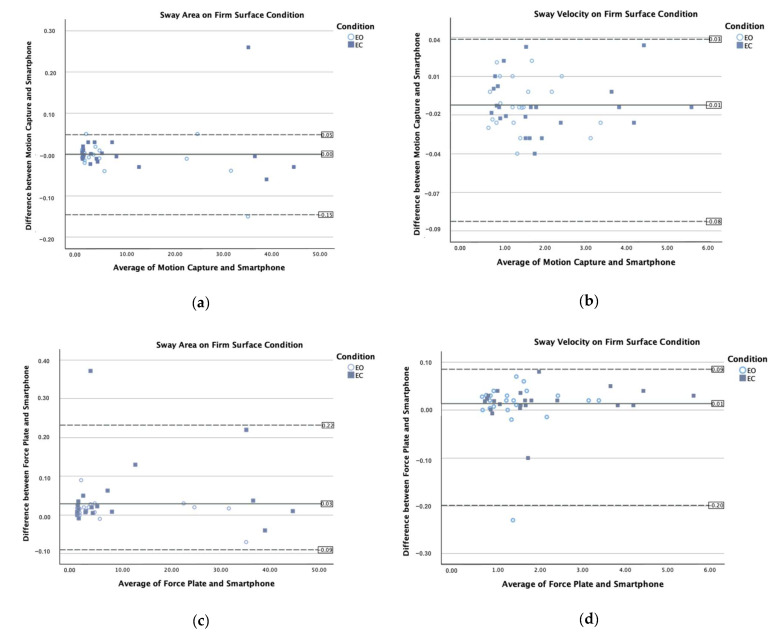

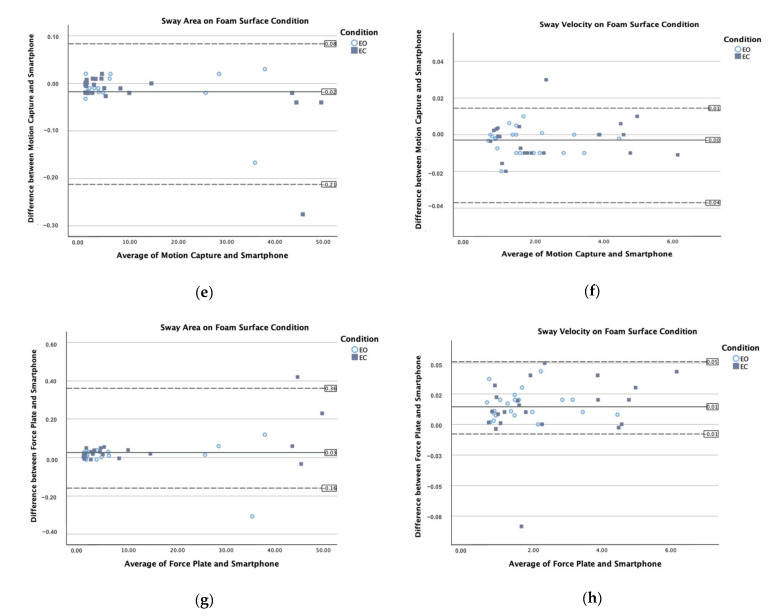
Bland–Altman plots of the mean of the measurements of the smartphone and each gold-standard instrument (motion capture and force plate) against the difference in the measurement of individual participants for the sway area (left: **a**,**c**,**e**,**g**) and sway velocity (right: **b**,**d**,**f**,**h**) for each visual and surface condition.

Bland–Altman plots for the sway area and sway velocity illustrated minimal measurement errors between the smartphone and each of the gold-standard methods (see Figure 5). The plots revealed that the mean differences between the smartphone and the reference devices were close to zero, indicating that the smartphone provided measurements that were largely in agreement with the gold-standard instruments. Furthermore, the limits of agreement (LOAs) were narrow, with only two outliers, suggesting that the variation between the two measurement methods was consistently small.

**Figure 6:**
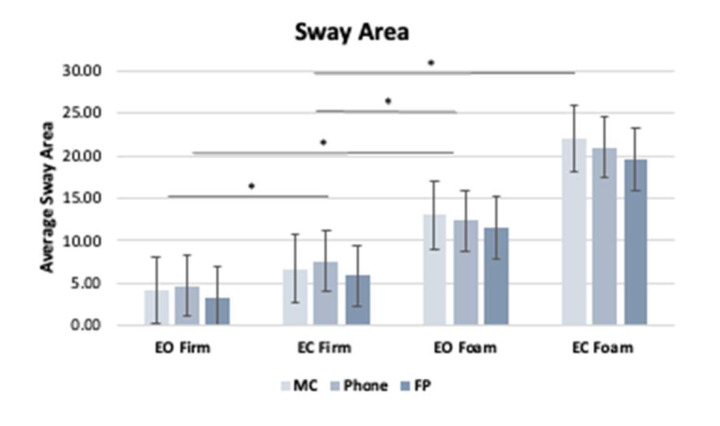

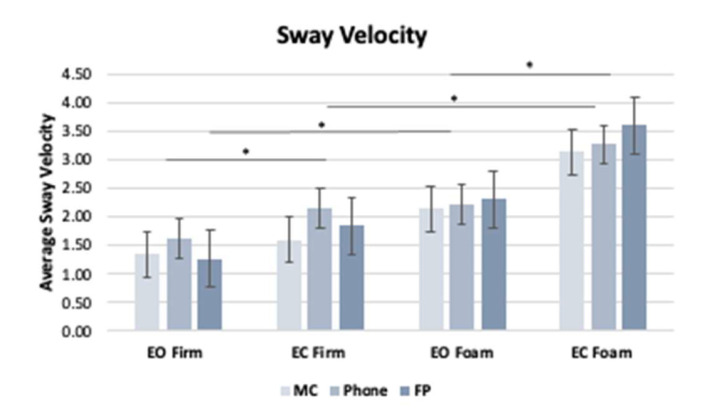
Comparison of synchronously collected data from the smartphone, motion capture, and force plate systems in each visual and surface condition for the postural outcome measures. Note asterisks (*) denotes statistically significant differences between visual and surface conditions.

When the postural data from the smartphone, motion capture, and force plate systems were collected synchronously (see Table 3), no significant differences (*p* > 0.05) were found between the devices for the sway area and sway velocity, irrespective of the visual and surface conditions (see Table 4 and Figure 6). However, the results for the postural outcome of the sway area indicate a main effect of the visual condition (EO vs. EC) and a main effect of the surface condition (Firm vs. Foam). Similar findings were observed in the sway velocity.

The test–retest reliability across repeated trials (*n* = 3) for each device showed excellent reliability (ICC (3,1) > 0.90; *p* < 0.001) for each of the visual/surface conditions (Table 5). Moderate-to-excellent reliability was found (ICC (3,*k*) = 0.60–0.97; *p* < 0.01) when comparing the somatosensory ratios from the smartphone app to the MC or the FP (Table 6). Primarily good-to-excellent reliability was observed for each device when analyzing the average sway area (ICC (3,*k*) = 0.94–0.99; *p* < 0.001) or average sway velocity (ICC (3,*k*) = 0.84–0.94; *p* < 0.001) in all of the visual and surface conditions (Table 7).

## 4. Discussion

The primary aims of this study were to investigate the validity, intersession reliability, and sensitivity of a novel smartphone application by testing it under different postural test conditions and comparing it to multiple validated research-grade posturography instruments. Given the widespread ownership of cellphones in the US general population [18], a smartphone device that could be used easily to objectively measure balance could offer an additional option of accessible healthcare that traditional clinical and research settings simply cannot provide. To our knowledge, this is the first study to cross-validate a custom postural smartphone application to both force plate COP and motion capture system COM postural data synchronously. Our current findings provide both criterion and construct validation for our novel smartphone application.

To address the validity, postural outcome measures, including the sway area and sway velocity, were compared during a range of visual and surface conditions administered to each participant to gain knowledge of the limitations of the smartphone when compared to research-grade gold standards. When compared, the smartphone application demonstrated strong, positive correlations under conditions with small (EO on a firm surface) and larger (EC on a foam surface) postural movements (Figure 4). As the postural conditions became more challenging and the sway area and sway velocity increased, the r-values increased. Additionally, time-series correlations (Table 2) were employed to assess the relationship between the motion capture system and smartphone, analyzing both the AP and ML axes. Moderate-to-high correlations were observed in both axes, indicating a relationship not only in terms of the average across an entire trial, but also changes in the spatiotemporal data. Interestingly, the ML axis, while statistically significant, proved to be less sensitive when collecting more stable (smaller postural moments) versus more challenging conditions (i.e., EO–Firm vs. EC–Foam). The findings align with the postural outcome measure correlations. This suggests that the threshold sensitivity was exceeded, and the smartphone was better able to detect the postural adjustments accurately. A plausible reason for this may be due to the design functionality of smartphone sensors, which is for day-to-day usability to capture larger movements during walking, fitness, gaming, and general user experience. However, these types of movements are not meant to test the lower limits of the smartphone IMUs. By altering the level of difficulty of the various standing tasks and eliciting a range of postural responses, the current study gained insight into whether the smartphone is capable of being a stand-alone device. No matter the surface or visual condition (Figure 6), there was no statistically significant difference in the postural measurements among the devices.

The novel application indicated a good-to-high degree of inter-rater reliability compared to each gold-standard device when analyzing the sway area and sway velocity averages for each visual and surface condition, as well as a moderate-to-good degree of reliability between the custom smartphone application and each gold-standard devices’ somatosensory ratios. This affirms the smartphone’s ability to measure data consistently when compared to two widely used gold-standard devices in posturography. Additionally, there was a high degree of intra-rater reliability among the trials (n = 3) within each device. Demonstrating both inter-rater and intra-rater reliability against gold-standard measures and itself is essential for the smartphone application to be accepted and trusted by clinicians and researchers. This reliability ensures consistent results, which are vital for accurate diagnoses and treatment plans. Furthermore, it promotes standardized practices in healthcare settings and home use, ultimately enhancing patient care and quality.

To examine the sensitivity of the device for detecting changes in balance as the task difficulty changes, we compared the output of the novel smartphone application between several conditions, in which one or both of the visual and somatosensory feedback were altered. The results demonstrated that our novel application was sensitive to detecting balance alterations during challenging visual (EC condition) and SOM (standing on a foam surface) feedback. As the application is sensitive to a large range of postural movements, it allows for the potential for individuals to track postural decline over time. This is especially important in clinical settings, where many conditions include the progressive deterioration of the postural control systems, thus decreasing the balance and increasing the fall risk. Clinicians often must rely on their patients’ subjective recollection concerning past falls and balance instability. By using objective and trackable assessments, clinicians will be better informed with the critical information necessary to shape proper treatment plans to reduce falls and prevent chronic complications associated with poor balance. Our proposed application provides a convenient, portable, and easy-to-use instrument that has the potential to be used at home or in any clinical setting without the initial need for a specialist. While it is not meant to be a diagnostic tool, it can serve as a screening tool that can be coupled with other assessments to enhance clinical judgment.

Several factors differentiate our custom application from other studies on smartphone IMU use for postural measurements. Previous studies have used smartphone devices to investigate various postural, functional, and gait movements that traditionally elicit larger postural movement (i.e., single-leg stance and gait) when reporting the validity of the phone [14,15,22,28,43]. However, few studies validate smartphone technology to gold-standard devices for smaller movements (i.e., quiet stance), and those that do were complementary to the present study. Additionally, from a methodological standpoint, the use of the belt in conjunction with using an electronic level to verify the phone orientation offers advantages, particularly during the validation process. The standardization of a rigorous set-up process enhances the quality of the data of the smartphone’s IMUs, contributing to increased correlations and the reliability of the smartphone across different trials and devices. Interestingly, the majority of studies investigating smartphone IMUs and balance do not note misalignments between the phone and the gravity vector due to any tilt of the device [36], particularly in multiple studies involving devices held by a human or in larger movements where the phone is not securely attached to the individual, which may cause extraneous movements of the device. This methodology cannot standardize the orientation and tilt of the phone [12]. This variability may introduce additional noise into the signal, potentially compromising the data. By changing the orientation of the phone, the current study was able to increase the signal-to-noise ratio, enhancing the reliability and sensitivity of the data. More research will need to be performed to understand the design of the IMUs with respect to the sensitivity of each axis in capturing smaller postural movements.

The current study’s methodology creates a minimal-risk task that can be administered both in a clinical setting but also in the comfort of one’s home, which is a primary long-term goal of the present application. Challenging tasks used to validate smartphones in previous studies that elicit postural responses were not suitable for an at-home setting, as they require a spotter and can cause an increased fall risk, especially in populations with compromised postural control. The current study was able to utilize a minimal-risk postural task when using the smartphone application without sacrificing the potential safety of the person in an at-home setting.

While this study provides valuable evidence for the use of this proposed application in clinical and at-home settings, it is important to acknowledge its limitations. A belt was used to secure the smartphone in a specific orientation during data collection to increase the sensitivity of the IMUs. Additionally, for the duration of the study, a single iPhone 14 was used for all 22 instances of data collection. Future studies should explore the intra-device reliability to generalize the application across smartphone models. Additionally, the current application was designed solely for Apple’s iOS. Further development will be required to determine whether a solution that is platform-agnostic is possible, thus enabling the application to work independently of a smartphone operating system.

## 5. Conclusions

The purpose of this study was to develop a novel smartphone application and validate it relative to research-grade posturography instrumentation (i.e., motion capture and force plate systems). By assessing the validity, sensitivity, and accuracy of a custom-designed smartphone application for measuring balance in healthy adults, we aimed to bridge the gap between at-home and clinical care. The results show strong agreement with established gold standards in posturography, supporting the application’s criterion validity. Additionally, the application demonstrated construct validity by being sensitive to changes in postural stability as the visual and/or surface condition was altered, indicating good intra-rater reliability. By leveraging smartphone sensors, the application offers the potential for remote balance monitoring and improved patient engagement, which is both critical for the early detection and management of fall risk, especially in populations with limited access to preventive care.

## Figures and Tables

**Table 1:** Participant demographics (N = 22).

Age (mean ± sd; range)	43.0 ± 13.1 yrs.; 20–60 yrs.
Gender	M: 11; F: 11
Height	66.4 ± 4.8 inches
Weight	180.2 ± 40.8 pounds

**Table 2:** Average Pearson correlations across the participants comparing positional time series data from the smartphone position to the motion capture system for the AP and ML axes.

		MC vs. Phone	
Condition (Vision, Surface)		AP Position	ML Position
EO, Firm	r	0.857	0.397
	*p*	<0.001	0.003
EC, Firm	r	0.886	0.432
	*p*	<0.001	0.002
EO, Foam	r	0.893	0.693
	*p*	<0.001	<0.001
EC, Foam	r	0.913	0.707
	*p*	<0.001	<0.001

Note: 22 participants per condition and 3 trials per participant; *N* = 3000 per trial.

**Table 3:** Mean ± standard deviation of the dependent variables, the sway area (cm^2^) and sway velocity (cm/s) (N = 22), for each test condition per device.

	Device	Mean ± Standard Deviation
Sway Area (EO), Firm	MC	4.11 ± 4.1
	Phone	4.61 ± 4.1
	FP	3.35 ± 4.5
Sway Area (EC), Firm	MC	6.65 ± 9.0
	Phone	7.56 ± 8.4
	FP	5.87 ± 9.1
Sway Velocity (EO), Firm	MC	1.35 ± 1.0
	Phone	1.62 ± 0.8
	FP	1.27 ± 0.9
Sway Velocity (EC), Firm	MC	1.60 ± 1.1
	Phone	2.25 ± 1.2
	FP	1.84 ± 1.2
Sway Area (EO), Foam	MC	13.03 ± 9.03
	Phone	12.38 ± 8.2
	FP	10.54 ± 9.4
Sway Area (EC), Foam	MC	22.02 ± 12.9
	Phone	20.95 ± 12.8
	FP	17.54 ± 12.9
Sway Velocity (EO), Foam	MC	4.11 ± 4.1
	Phone	4.61 ± 4.1
	FP	3.35 ± 4.5
Sway Velocity (EC), Foam	MC	6.65 ± 9.0
	Phone	7.56 ± 8.4
	FP	5.87 ± 9.1

**Table 4:** Summary table of the repeated-measures analysis of variance (rmANOVA) for the dependent variables.

		df				
	Variable	Between Groups	Within Groups	F	Sig.	Partial Eta Squared (η^2^)
Sway Area	Surface Condition	1	21	49.54	<0.001	0.70
	Device	1.76	36.89	6.83	0.08	0.25
	Visual Condition	1	21	30.35	<0.001	0.59
Sway Velocity	Surface Condition	1	21	93.7	<0.001	0.82
	Device	1.69	35.62	2.84	0.07	0.12
	Visual Condition	1	21	46.06	<0.001	0.69

Device degrees of freedom were adjusted using a Greenhouse–Geisser correction.

**Table 5:** Test–retest reliability was evaluated using an ICC (3,1) 2-way mixed-effects model, absolute agreement and their 95% confidence intervals were calculated to evaluate repeated trials (n = 3) within each device.

		ICC (3,1) (95% Confidence Interval)	
Device	Visual, Surface Condition	Sway Area	Sway Velocity
MC	EO, Firm	0.952 (0.903–0.978)	0.982 (0.963–0.992)
	EC, Firm	0.964 (0.926–0.984)	0.982 (0.943–0.987)
	EO, Foam	0.904 (0.784–0.952)	0.957 (0.888–0.983)
	EC, Foam	0.953 (0.703–0.964)	0.960 (0.908–0.983)
Phone	EO, Firm	0.930 (0.857–0.969)	0.978 (0.956–0.990)
	EC, Firm	0.964 (0.927–0.984)	0.977 (0.954–0.990)
	EO, Foam	0.909 (0.815–0.959)	0.916 (0.818–0.963)
	EC, Foam	0.902 (0.802–0.956)	0.951 (0.895–0.978)
FP	EO, Firm	0.900 (0.797–0.955)	0.979 (0.957–0.990)
	EC, Firm	0.962 (0.922–0.983)	0.976 (0.950–0.989)
	EO, Foam	0.968 (0.935–0.986)	0.994 (0.986–0.998)
	EC, Foam	0.945 (0.890–0.975)	0.968 (0.933–0.986)

**Table 6:** ICC (3,k) estimates and their 95% confidence intervals were calculated based on a mean-rating (k = 3), absolute-agreement, 2-way mixed-effects model for the somatosensory ratio calculated using the sway area or sway velocity for the novel device when compared to the motion capture or force plate systems.

		ICC (3,*k*) (95% Confidence Interval)	
Postural Outcome Measure	Surface Condition	MC vs. Phone	FP vs. Phone
Sway Area	Firm	0.753 (0.419–0.897)	0.729 (0.334–0.888)
	Foam	0.826 (0.583–0.927)	0.839 (0.615–0.933)
Sway Velocity	Firm	0.715 (0.297–0.883)	0.598 (0.038–0.833)
	Foam	0.968 (0.923–0.987)	0.828 (0.460–0.936)

**Table 7:** ICC (3,*k*) estimates and their 95% confidence intervals were calculated based on a mean-rating (*k* = 3), absolute-agreement, 2-way mixed-effects model for measurement outcome (sway area and sway velocity) averages for the novel device, and motion capture and force plate systems.

		ICC (3,*k*) (95% Confidence Interval)
Postural Outcome Measure	Visual, Surface Condition	
	EO, Firm	0.959 (0.908–0.982)
Sway Area	EC, Firm	0.989 (0.973–0.996)
	EO, Foam	0.948 (0.892–0.977)
	EC, Foam	0.940 (0.871–0.974)
	EO, Firm	0.842 (0.682–0.929)
	EC, Firm	0.935 (0.804–0.975)
Sway Velocity	EO, Foam	0.894 (0.786–0.953)
	EC, Foam	0.899 (0.795–0.955)

## Data Availability

The raw data supporting the conclusions of this article will be made available by the authors on request.
